# [Retracted] Knockdown of ubiquitin-specific protease 51 attenuates cisplatin resistance in lung cancer through ubiquitination of zinc-finger E-box binding homeobox 1

**DOI:** 10.3892/mmr.2025.13718

**Published:** 2025-10-20

**Authors:** Feng Zhou, Chunling Du, Donghui Xu, Jinchang Lu, Lei Zhou, Chaomin Wu, Bo Wu, Jianan Huang

Mol Med Rep 22: 1382–1390, 2020; DOI: 10.3892/mmr.2020.11188

Following the publication of the above paper, it was drawn to the Editor's attention by a concerned reader that the control GAPDH western blotting data shown in Fig. 2B on p. 1385 were strikingly similar to data appearing in different form in another article written by different authors at different research institutes that had already been published in the journal *Biomedicine & Pharmacotherapy*. Certain of the other western blot data featured in Figs. 2 and 4 were likewise very similar to data which subsequently appeared in other articles that were unrelated to this one.

In view of the fact that the abovementioned data in Fig. 2C had already apparently been published previously, the Editor of *Molecular Medicine Reports* has decided that this paper should be retracted from the Journal. The authors were asked for an explanation to account for these concerns, but the Editorial Office did not receive a reply. The Editor apologizes to the readership for any inconvenience caused.

